# Diet Quality is Positively Associated With Nature Relatedness in a U.S. Population: A Pilot Study

**DOI:** 10.1016/j.pmedr.2024.102924

**Published:** 2024-11-05

**Authors:** Dahlia Stott, Jonathan M. Deutsch, Micheal Bruneau, Jennifer A. Nasser, Mara Z. Vitolins, Brandy-Joe Milliron

**Affiliations:** a Department of Health Sciences, Drexel University, 60 N 36^th^ Street, Philadelphia, PA, USA; b Department of Health Sciences, Drexel University, 101 N 33^rd^ Street, Philadelphia, PA, USA; c Wake Forest University School of Medicine, 1 Medical Center Blvd, Winston-Salem, NC, USA

**Keywords:** Diet quality, HEI-2020, Nature Relatedness, Connection to nature, Dietary intake

## Abstract

**Background:**

Sustainable dietary practices can help reduce greenhouse gas emissions and promote planetary health. The importance of investigating how to promote sustainable dietary practices is therefore crucial. Nature Relatedness measures an individual’s connection to nature and can predict environmental concern and stewardship. While emerging research has suggested those with a higher degree of Nature Relatedness report the intention to follow more sustainable dietary practices, the relationship between actual dietary intake and Nature Relatedness has yet to be fully explored. Therefore, the purpose of this pilot study was to assess the relationship between diet quality and Nature Relatedness.

**Methods:**

In this cross-sectional study, participants across the United States completed the online survey from September to November 2023. Diet History Questionnaire II and Nature Relatedness scale were completed by the participants. Diet quality was assessed using the Healthy Eating Index (HEI)-2020 total and component scores. Nature Relatedness total and subscale scores were computed. Pearson and Spearman Rho correlation coefficients assessed associations between HEI-2020 and Nature Relatedness. Simple linear regression models examined the relationships between HEI-2020 total and component scores and Nature Relatedness (controlling for age, gender, race, and education).

**Results:**

Three hundred adults completed the study. HEI-2020 total score was positively associated with Nature Relatedness (*p* < 0.001). Total fruit, total vegetable, green and beans, and refined grains component scores were positively associated with Nature Relatedness (*p* < 0.001). Nature Relatedness significantly predicted diet quality and total fruit, total vegetable, greens and beans, and moderation of refined grains consumption.

**Conclusions:**

There are positive and significant relationships between diet quality, components of dietary intake that may promote planetary health, and Nature Relatedness. Our findings may be used to inform future research and nutrition intervention programs promoting personal and planetary health through nature-based interventions.

## 1 Introduction

Food contributes to approximately one-third of green-house gas emissions caused by humans, including but not limited to production, transportation, and waste. (Crippa et al., 2021) In an effort to lessen the impact of food on greenhouse gas emissions, the Food and Agriculture Organization of the United Nations and the Food Climate Research Network have recommended that sustainable practices be integrated into dietary guidelines across the world, as current guidelines that prioritize human health potentially jeopardize planetary health. (Fischer CG, Garnett T. Plates, Pyramids, Planet Developments in National Healthy and Sustainable Dietary Guidelines: A State of Play Assessment. Food and Agriculture Organization of the United Nations The Food Climate Research Network at The University of Oxford;, 2016) To address these concerns, the EAT-Lancet commission and other organizations have created their own sets of dietary recommendations, which generally are to follow a plant forward diet that includes greater intake of fruits, vegetables, and whole grains, and limit intake of animal products and added sugars. (Willett et al., 2019, Brink et al., 2019) Most of these dietary components are emphasized in the Dietary Guidelines for Americans (DGA) and measured by the Healthy Eating Index-2020 (HEI-2020). (U.S. Department of Agriculture, U.S. Department of Health and Human Services. Dietary Guidelines for Americans, 2020, Shams-White et al., 2023) Blackstone and Conrad (2020) compared the EAT-Lancet commission’s recommended diet and the DGA, finding that the DGA’s total vegetable, poultry, seafood, eggs, beans and peas, dairy, and unsaturated fatty acid recommendations were within the ranges recommended by the EAT-Lancet commission. (Blackstone and Conrad, 2020) The DGA’s fruit recommendation exceeds that of the EAT-Lancet commissions by 21 %, while the EAT-Lancet commission recommends whole grains 2.3 times the amount that the DGA recommends. (Blackstone and Conrad, 2020) Given the impact that positive dietary habits have on preventing adverse personal and planetary health, it is important to understand how sustainable diets can be fostered in people’s lives.

Connection to nature is a person’s relationship with nature based on their values and beliefs about nature and attitudes and behaviors when in nature; (Salazar et al., 2021) it has been used to understand an individual’s environmental concern and stewardship. Behaviors associated with connection to nature have included being a part of an environmental organization, self-identification as an environmentalist, avoiding waste, recycling, concern about climate change, and engagement in nature-based activities. (Whelan et al., 2022, Whitburn et al., 2020, Nisbet et al., 2008, Selinske et al., 2023) Researchers have demonstrated that there are relationships among connection to nature, the intention to eat sustainably, the purchasing of organic, climate friendly, seasonal, and local foods. (Weber et al., 2020, Spendrup et al., 2016, Teixeira et al., 2023) Limited research has explored the relationship between dietary consumption and connection to nature. Milliron et al. (2022) were the first to report that Nature Relatedness (NR), a measurement of connection to nature, was associated with fruit and vegetable consumption and dietary diversity in an urban population. (Milliron et al., 2022).

Notwithstanding, more research is needed to understand how connection to nature is associated with dietary intake. Therefore, the purpose of the current study was to assess the relationship between diet quality, as measured by HEI-2020, and Nature Relatedness. Results from this pilot study may be used to inform future research and dietary programs promoting personal and planetary health through nature-based interventions.

## 2 Methods

### 2.1 Study Design & population

In this pilot cross-sectional analysis, participants across the United States completed an online survey via Qualtrics® (Qualtrics, Provo, UT, USA). Individuals were recruited from ResearchMatch® and digital advertisements via Build Clinical. ResearchMatch® is a national health registry of individuals who are interested in participating in research studies. Build Clinical is a recruitment service that runs online advertisement campaigns to recruit for research studies. Individuals who were interested in participating in the study completed a screening survey on REDCap (ResearchMatch®) or the study landing page (Build Clinical).

Participants were eligible if they were over 18 years of age, had at least a high school education, were fluent in English, resided in the United States, and had access to a computer and internet. Once participants were determined to be eligible, they reviewed the consent form and provided consent online as proxy for written informed consent. Participants received $10 for completing the study. Data collection occurred from September to November 2023. Review and approval for the study along with all procedures were obtained from the Institutional Review Board (IRB) at Drexel University.

### 2.2 Diet quality

Participants completed the Diet History Questionnaire II, a semi-quantitative food frequency questionnaire that asks individuals about their dietary intake over the previous month. (Questionnaire, 2010) In the Diet History Questionnaire II, the participants were asked about their consumption of 134 food items and eight supplements. This version of the assessment was chosen for the current study because the paper version could be integrated into the Qualtrics® survey. HEI-2020 scores were calculated with Diet*Calc (National Cancer Institute, Silver Spring MD) and SAS® 9.4 (SAS Institute, Cary NC).

HEI-2020 was used to measure dietary quality and assesses individuals’ adherence to the DGA, 2020–2025 edition. (Shams-White et al., 2023) The index scores components that should be consumed adequately and moderately. The adequacy components are total fruits, whole fruits, total vegetables, greens and beans, whole grains, dairy, total protein foods, seafood and plant proteins, and fatty acids. The moderation components are refined grains, sodium, added sugars, and saturated fats. Each component has a standard minimum score of 0 and a maximum score of 5 or 10; higher component scores indicate greater adherence to dietary guidelines. The component scores are summed to calculate the total score. The total score ranges from 0 to 100 and a greater score reflects greater adherence to the DGA. For the purpose of the current investigation, HEI-2020 components that are aligned with sustainable dietary practices include total fruits, whole fruits, total vegetables, greens and beans, whole grains, seafood and plant proteins, and moderation of refined grains. (Willett et al., 2019).

### 2.3 Nature Relatedness

The NR scale was used to assess participants’ connection to nature. (Nisbet et al., 2008) This scale comprises three subscales: Self, Perspective, and Experience. (Nisbet et al., 2008) NR Self measures an individuals’ personal connection to nature (e.g., I always think about how my actions affect the environment). NR Perspective assesses an individual’s external world view of nature (e.g., The state of non-human species is an indicator of the future for humans.). NR Experience measures an individual's physical familiarity with nature (e.g., I enjoy digging in the earth and getting dirt on my hands). Participants rated their agreement of 21 items via a 5-item Likert scale. Certain items were reverse scored and then all the items were averaged to calculate the total and subscale scores (Self, Perspective, and Experience). Scores range from 0 to 5 and a greater score indicates greater connection to nature. The NR scale has demonstrated acceptable validity. (Nisbet et al., 2008).

### 2.4 Data analysis

Data analysis was conducted using IBM SPSS Statistics, version 29.0 (Armonk, NY: IBM Corp). Participant demographics were tabulated as frequencies and percentages. Mean and median were calculated for HEI-2020 and NR scores. Normally distributed data are displayed as mean (SD) and non-normally distributed data are displayed as median (IQR). There was no missing data. Pearson correlation was used to assess the relationship between HEI-2020 total score and NR total score. Spearman Rho tests were used to assess the relationships between HEI-2020 total and component scores and NR total and subscale scores. Linear regression models were used to assess the predictive relationship between HEI-2020 total, total fruit, total vegetable, green and bean, and refined grain component scores and NR, adjusting for age, gender, race, and education.

## 3 Results

Participant (n = 300) demographics are displayed in Supplemental Table 1. Participants had a mean (SD) HEI-2020 total score of 62.87 (9.85) and NR score of 3.64 (0.61). Diet quality was positively associated with NR total (*p* < 0.001) and subscale scores: Self (*p* < 0.001), Perspective (*p* < 0.001), and Experience (*p* < 0.001). Total fruit was positively associated with NR total (*p* < 0.001), Self (*p* < 0.001), and Experience (*p* < 0.05). Total vegetable was positively associated with NR total (*p* < 0.001) and subscale scores: Self (*p* < 0.001), Perspective (*p* < 0.001), and Experience (*p* < 0.001). Greens and beans was positively associated with NR total and subscale (Self, Perspective, and Experience) scores: Self (*p* < 0.001), Perspective (*p* < 0.001), and Experience (*p* < 0.001). Whole grain was positively associated with NR Perspective (*p* < 0.001). Moderation of refined grains was positively associated with NR total (*p* < 0.001), Self (*p* < 0.001), and Experience (*p* < 0.001). Table 1 displays average HEI-2020 and NR scores and correlation coefficients.

**Table 1:** Mean, standard deviation, median, interquartile range and correlation table of Healthy Eating Index-2020 (HEI-2020) total and component scores and Nature Relatedness (NR) total and subscale scoresa.

		NR Total	NR Self	NR Perspective	NR Experience
		3.64 (0.61)^b^	3.89 (0.89)^c^	3.83 (1.17)^c^	3.33 (1.17)^c^
HEI-2020 total	62.87 (9.85)^b^	0.26**	0.19**	0.22**	0.21**
Total fruits	4.22 (2.51)^c^	0.15**	0.21**	0.00	0.14*
Whole fruits	5.00 (0.00)^c^	0.04	0.08	−0.02	0.03
Total vegetable	4.38 (1.71)^c^	0.27**	0.22**	0.20**	0.21**
Greens and beans	4.74 (2.66)^c^	0.26**	0.14**	0.27**	0.19**
Whole grains	1.80 (1.71)^c^	0.11	0.01	0.14**	0.05
Dairy	4.45 (4.29)^c^	0.07	0.01	0.11	0.03
Total protein	5.00 (0.39)^c^	0.03	0.03	0.04	0.04
Seafood & plant protein	5.00 (1.46)^c^	0.08	0.04	0.11	0.06
Fatty acids	5.44 (4.16)^c^	−0.01	−0.01	0.03	−0.02
Sodium	4.59 (4.43)^c^	0.00	0.00	0.00	−0.04
Refined grains	8.11 (3.99)^c^	0.24**	0.19**	0.10	0.25**
Saturated fat	6.17 (4.42)^c^	0.02	0.05	−0.05	0.05
Added sugar	7.98 (3.82)^c^	0.08	0.01	0.09	0.11

a HEI-2020 total score and NR total score assessed with Pearson correlations. All other correlations are assessed with Spearman Rho.

b Mean (Standard deviation).

c Median (Interquartile rage).

* Significant at *p* < 0.05.

** Significant at *p* < 0.001.

Several linear regressions were conducted with NR as the predictor and HEI-2020 total and component scores as the outcomes. A significant regression with HEI-2020 total score was found (*F*(5, 294) = 10.01, *p* < 0.001), with an adjusted R^2^ of 0.13. A significant regression with total fruit was found (*F*(5, 294) = 3.10, *p* = 0.01), with an adjusted R^2^ of 0.03. A significant regression with total vegetable was found (*F*(5, 294) = 4.93, *p* < 0.001), with an adjusted R^2^ of 0.06. A significant regression with greens and beans was found (*F*(5, 294) = 4.47, *p* = 0.001), with an adjusted R^2^ of 0.05. A significant regression with moderation of refined grains was found (*F*(5, 294) = 12.64, *p* < 0.001), with an adjusted R^2^ of 0.16. For every point increase in NR, HEI-2020 score increases 3.11 points (*t* = 3.34, *p* < 0.001), total fruit score increases 0.44 points (*t* = 3.04, *p* < 0.01), total vegetable increases 0.44 points (*t* = 4.07, *p* < 0.001), greens and beans increases by 0.52 points (*t* = 3.24, *p* = 0.001), and moderation of refined grains increases 0.63 points (*t* = 2.64, *p* < 0.009) (Table 2).

**Table 2:** Linear regression of Nature Relatedness predicting Healthy Eating Index-2020 (HEI-2020) total and component scores.

				95 % CI		
	B	SE B	β	LL	UL	*p*
**Total HEI-2020**						
Crude model	4.17	0.90	0.26	2.40	5.94	< 0.001
Adjusted model^a^	3.11	0.91	0.19	1.33	4.90	< 0.001
**Total fruit**						
Crude model	0.43	0.14	0.18	0.15	0.70	< 0.01
Adjusted model^a^	0.44	0.14	0.18	0.16	0.72	0.003
**Total vegetable**						
Crude model	0.46	0.10	0.25	0.25	0.66	< 0.001
Adjusted model^a^	0.44	0.11	0.24	0.23	0.66	< 0.001
**Greens and beans**						
Crude model	0.60	0.15	0.22	0.30	0.90	< 0.001
Adjusted model^a^	0.52	0.16	0.19	0.20	0.83	0.001
**Refined grains**						
Crude model	1.06	0.24	0.25	0.59	1.54	< 0.001
Adjusted model^a^	0.63	0.24	0.15	0.16	1.11	0.009

a Adjusted for age, gender, race, and education.

## 4 Discussion

In this pilot study, diet quality and NR had a positive significant relationship and NR significantly predicted diet quality. Further, certain components of diet quality that may promote planetary health (namely total fruit, total vegetable, greens and beans, whole grain, and moderation of refined grains) (Willett et al., 2019) were positively correlated with NR total and subscale scores. It’s important to note that the following commentary about how our findings relate to planetary health is based on our assumptions and the literature, as we did not include metrics of planetary health in this analysis.

Some of our findings are aligned with the EAT-Lancet commission’s recommendations which encourages greater consumption of fruits, vegetables, and whole grains to promote both personal and planetary health. (Willett et al., 2019) Herein, we report that the corresponding dietary components in HEI-2020 are positively and significantly related to NR. While the whole grain component score is significantly correlated with NR Perspective, moderation of refined grains is significantly related to NR total, Self, and Experience. This highlights that consumption of dietary components that likely promote personal and planetary health are significantly related to an individual's connection to nature.

Our findings primarily confirm and expand upon the existing literature about dietary intake and people’s environmental concerns. (Milliron et al., 2022, Alhothali et al., 2021, Korkala et al., 2014, Slotnick et al., 2024) We previously reported that among an urban population, fruit and vegetable consumption is significantly related to NR total, Self and Experience component scores. (Milliron et al., 2022) In the present study, we add to this previous work by reporting that vegetable consumption is positively correlated with NR Perspective. Consistently across the literature, individuals who report greater consumption of foods that are considered “climate friendly” such as fruits and vegetables also report making dietary choices for the environment and being concerned about the environment and climate change. (Alhothali et al., 2021, Korkala et al., 2014, Slotnick et al., 2024).

Increasing people’s NR may promote their dietary habits for personal and planetary health. As previously described, NR is comprised of one’s personal connection (e.g., emotions and feelings) to nature, their external world view (e.g., knowledge and beliefs about nature), and their experiences in nature. (Nisbet et al., 2008) Zylstra et al. (2014) explain that providing culturally appropriate activities may provide people with the opportunity to increase their connection to nature. Examples of such activities recommended include interacting with or touching nature, cultivating wonder and awe in nature, participating in self-care when in nature (e.g., relaxing, physical activity), and becoming more familiar with wildlife. (Zylstra et al., 2014) These and other activities may be integrated into a nature-based program to promote sustainable dietary patterns.

Strengths of this study include utilizing validated and reliable measures of dietary intake and connection to nature. Despite these strengths, we acknowledge that our study is not without limitations. The cross-sectional nature of the study did not permit us to make assessments or declarations about causation. Food frequency questionnaires are also subjected to recall bias, which may have impacted what participants reported to have consumed over the previous month. In addition, there are other aspects of sustainable dietary patterns which were not explored in this analysis including but not limited to reducing food waste and consumption of local foods.

## 5 Conclusion

In this study, we found a positive and significant relationship between diet quality and NR. Further, consumption of dietary components that may promote planetary health are also associated with NR. Future research should continue to explore the interconnections between dietary intake, NR, and planetary health with a larger sample size. These findings could be used to promote dietary patterns for personal and planetary health by increasing NR through nature-based solutions.

## CRediT authorship contribution statement

**Dahlia Stott:** Writing – review & editing, Writing – original draft, Visualization, Software, Project administration, Methodology, Investigation, Formal analysis, Data curation, Conceptualization. **Jonathan M. Deutsch:** Writing – review & editing, Resources, Funding acquisition. **Micheal Bruneau Jr.:** Writing – review & editing. **Jennifer A. Nasser:** Writing – review & editing. **Mara Z. Vitolins:** Writing – review & editing. **Brandy-Joe Milliron:** Writing – review & editing, Supervision, Software, Resources, Methodology, Funding acquisition, Data curation, Conceptualization.

## Funding

This work was supported the Dean’s Rapid Response Relevant grant (R3), College of Nursing and Health Professions, Drexel University; and Summer Research Award, Drexel University.

## Declaration of competing interest

The authors declare that they have no known competing financial interests or personal relationships that could have appeared to influence the work reported in this paper.

## Data Availability

Data will be made available on request.
